# Nanometer-thick copper films with low resistivity grown on 2D material surfaces

**DOI:** 10.1038/s41598-022-05874-9

**Published:** 2022-02-02

**Authors:** Yu-Wei Liu, Dun-Jie Zhang, Po-Cheng Tsai, Chen-Tu Chiang, Wei-Chen Tu, Shih-Yen Lin

**Affiliations:** 1 Research Center for Applied Sciences, Academia Sinica, Academia Rd, No. 128, Sec. 2, Taipei, 11529 Taiwan; 2 Department of Electrical Engineering, National Cheng Kung University, No.1, University Road, Tainan City, 701 Taiwan; 3 Graduate Institute of Electronics Engineering, National Taiwan University, No. 1, Sec. 4, Roosevelt Rd, Taipei, 10617 Taiwan

**Keywords:** Electrical and electronic engineering, Materials for devices, Nanoscale materials

## Abstract

Thin Copper (Cu) films (15 nm) are deposited on different 2D material surfaces through e-beam deposition. With the assist of van der Waals epitaxy growth mode on 2D material surfaces, preferential planar growth is observed for Cu films on both MoS_2_ and WSe_2_ surfaces at room temperature, which will induce a polycrystalline and continuous Cu film formation. Relative low resistivity values 6.07 (MoS_2_) and 6.66 (WSe_2_) μΩ-cm are observed for the thin Cu films. At higher growth temperature 200 °C, Cu diffusion into the MoS_2_ layers is observed while the non-sulfur 2D material WSe_2_ can prevent Cu diffusion at the same growth temperature. By further increasing the deposition rates, a record-low resistivity value 4.62 μΩ-cm for thin Cu films is observed for the sample grown on the WSe_2_ surface. The low resistivity values and the continuous Cu films suggest a good wettability of Cu films on 2D material surfaces. The thin body nature, the capability to prevent Cu diffusion and the unique van der Waals epitaxy growth mode of 2D materials will make non-sulfur 2D materials such as WSe_2_ a promising candidate to replace the liner/barrier stack in interconnects with reducing linewidths.

## Introduction

In silicon (Si) industry, copper (Cu) has been widely used for the application of interconnects due to its low resistivity^1–3^. On the other hand, the high diffusivity of Cu will result in Cu atom diffusion into the dielectric layers such that short circuit and therefore, system failure will be observed for Si chips^4–7^. Therefore, to avoid the problem of Cu diffusion, diffusion barrier layers between Cu wires and dielectrics are required for Cu interconnects. In nowadays, thin TaN layers (~ 3 nm) are commonly adopted as the diffusion barrier layer to isolate the Cu wires (~ 20 nm in widths/heights) from the surrounding dielectrics^7,8^. For a better adhesion between Cu and the TaN diffusion barrier layer, an additional liner layer Ta (~ 1 nm) is also required^9^. In this case, the Cu/Ta/TaN stack has become the standard interconnect structure in modern semiconductor industries. However, with the further shrinkage in the device line widths below 3 nm, interconnect scaling down to sub-20 nm will be required in the future. With the reduction of Cu wire thicknesses, its resistivity will increase remarkably due to the electron scattering by phonons, point defects, impurities and grain boundaries^9–14^. To avoid significant resistivity increasing with reduced Cu wire thickness, an alternate choice would be the thinner barrier/liner layers. However, when the TaN film thickness is reduced to below 3 nm, the barrier layer is no longer able to prevent the Cu diffusion to the surrounding dielectrics^14^. In this case, a new material with the capability of blocking Cu diffusion in a few nanometer thicknesses will become an urgent need for further interconnect scaling. Under this scenario, since mono-layer 2D materials are of few angstroms in thicknesses, they will become a promising candidate for this application. Besides the thin body nature of 2D materials, in previous publications, we have demonstrated vertical 2D material hetero-structures with epitaxially grown elemental 2D materials such as antimony (Sb), germanium (Ge) and tin (Sn) grown on molybdenum disulfide (MoS_2_) surfaces^15,16^. The results have revealed that the epi-layer formation through the van der Waals epitaxy growth mode on 2D material surfaces has less dependent to the substrate lattice structures. By further expanding this concept to metal crystals, van der Waals epitaxy on 2D material surfaces will help to improve the crystallinity of thick gold (Au) films grown on MoS_2_ surfaces^17^. The same mechanism will also help to improve the continuity of the nanometer-thick Au film such that close-to-theory sheet resistance will be obtained for 10 nm Au film grown on MoS_2_ surfaces^18^. The continuous thin Au films grown on MoS_2_ surfaces have also demonstrated good adhesion and wettability at the metal/2D material interfaces. Therefore, by using the thin 2D materials to replace the Ta/TaN stack, further interconnect scaling may be achieved. The unique van der Waals epitaxy growth mode on 2D material surfaces will be advantageous for the formation of continuous metal films with improved crystallinity such that even lower resistivity values can be obtained.

In this paper, we have deposited 15 nm Cu films on MoS_2_ and WSe_2_ surfaces by using the e-beam deposition system. With the assist of van der Waals epitaxy growth mode, polycrystalline Cu films are obtained on 2D material surfaces at room temperature (RT). By using the non-sulfur 2D material such as WSe_2_, Cu diffusion can be avoided at higher growth temperatures. By further increasing the deposition rates, a record-low resistivity value 4.62 μΩ-cm for thin Cu films is observed for the sample grown on the WSe_2_ surface. The thin thicknesses down to few atomic layers and good wettability of WSe_2_ to Cu have made it a promising candidate to replace the liner/barrier stack in interconnects with reducing linewidths.

## Results and discussions

### Thin copper films grown on different substrates

**Figure 1:**
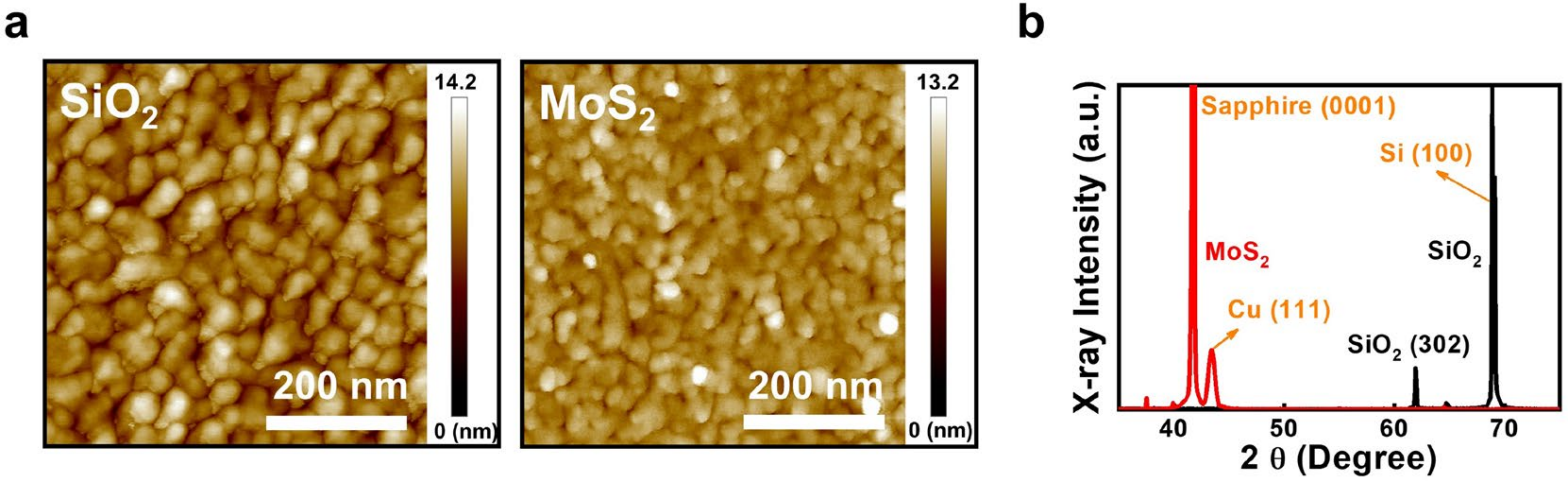
(**a**) The 500 × 500 nm^2^ AFM images and (**b**) The 2θ − θ curves measured by XRD of 15 nm Cu films deposited on 300 nm SiO_2_/Si and tri-layer MoS_2_/c-plane sapphire substrates at RT.

To investigate the influence of van der Waals epitaxy to the thin Cu films, 15 nm Cu were deposited on 300 nm SiO_2_/Si and tri-layer MoS_2_/c-plane sapphire substrates at RT by using a e-beam deposition system with 0.1 Å/s deposition rate. The 500 $\times$ 500 nm^2^ atomic force microscopy (AFM) images of the two samples are shown in Fig. 1a. As shown in the figure, Cu clusters with deep trenches are observed for the sample grown on the SiO_2_/Si substrate, which indicates that the dangling bonds on SiO_2_ surfaces may hinder the Cu atom migration and therefore, island formation instead of planar film growth will be observed on SiO_2_ surfaces. Different from the Cu film grown on SiO_2_ surfaces, a more continuous Cu film is observed on the MoS_2_ surface. Although Cu clusters are still observed for the sample, the shallower trenches between the clusters suggest that the van der Waals epitaxy growth mode on 2D material will help the planar growth of thin metal films. The root-mean-square (RMS) roughness values of the Cu films deposited on SiO_2_ and MoS_2_ surfaces are 1.67 and 1.04 nm, respectively, which is consistent with the observation that deeper trenches are observed for the Cu film deposited on the SiO_2_ surface. As a result, compared with the resistivity value 19.71 μΩ-cm for the sample grown on the SiO_2_/Si substrate, a much lower resistivity 6.07 μΩ-cm will be observed for the sample grown on the MoS_2_ surface, which is close to the ~ 5 μΩ-cm resistivity obtained from 15 nm Cu film grown on mechanically exfoliated MoS_2_ surfaces^19^. The 2θ − θ curves of the two samples measured by the X-ray diffraction system (XRD) are shown in Fig. 1b. Except for the substrate peaks, an additional Cu (111) peak is observed only for the Cu film grown on the MoS_2_ surface, which suggests that besides planar film growth, a better crystallinity is also obtained for the Cu film grown on MoS_2_ surfaces. The observation of Cu (111) peak alone may suggests that most of the Cu domains grown on MoS_2_ surfaces are well-aligned with (111) direction. Further investigation is required in the future to verify this point. On the other hand, it is expected that different orientations of the Cu domains are obtained on the SiO_2_ surface. Therefore, compared with the well-aligned (111) direction for the Cu domains on the MoS_2_ surface, the weakened XRD peaks of the Cu domains are not observed for the Cu film deposited on the SiO_2_ surface. The improved crystallinity of the Cu films grown on 2D material surfaces may help to reduce the inelastic electron scatterings on the Cu grain boundaries.

### Copper films grown on MoS_2_ surfaces at different temperatures

**Figure 2:**
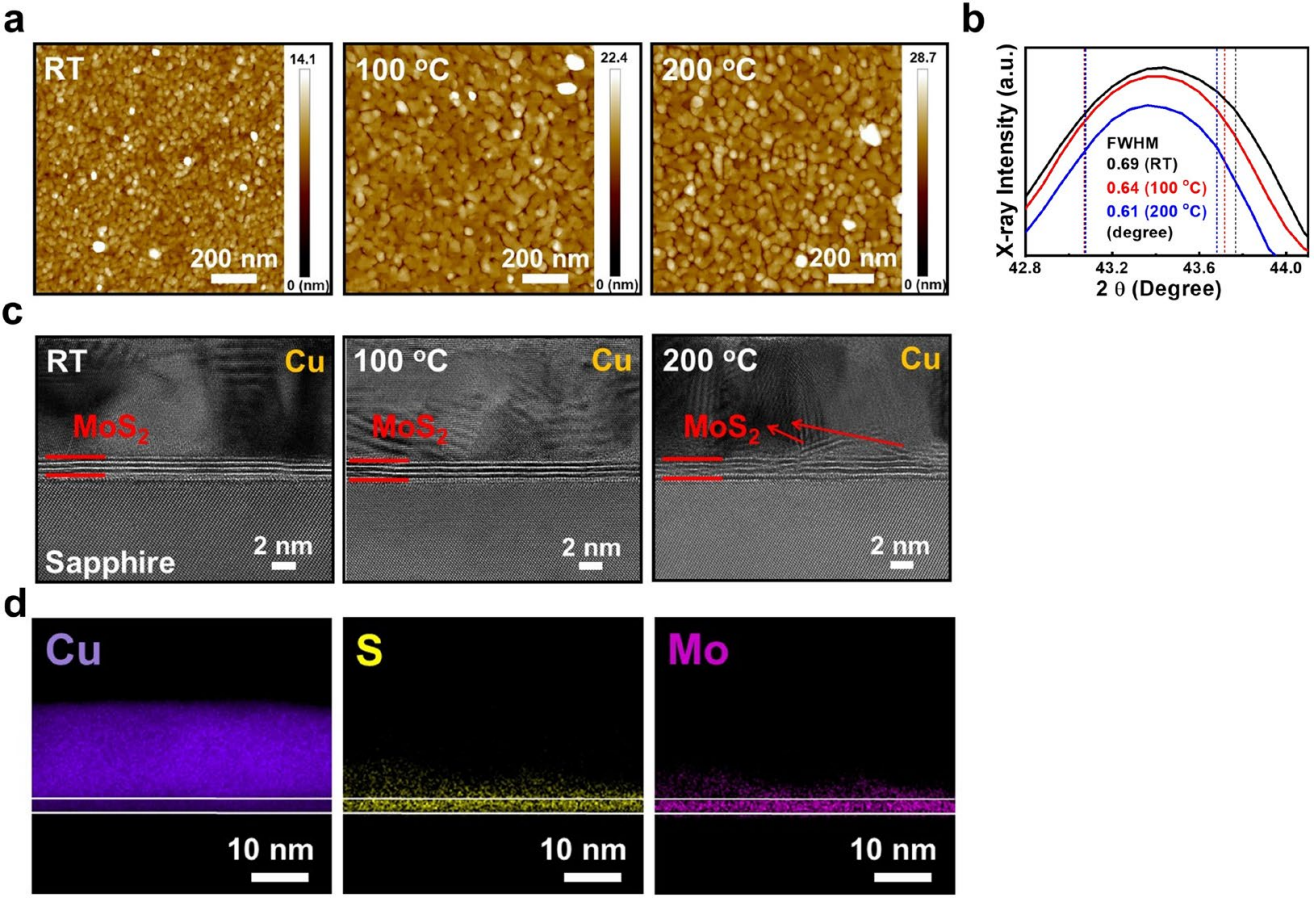
(**a**) The 1 × 1 $\upmu$m^2^ AFM images, (**b**) the Cu (111) XRD peak and (**c**) the cross-sectional HRTEM images of 15 nm Cu deposited on tri-layer MoS_2_/sapphire substrates at RT, 100 and 200 °C. (**d**) The HAADF mappings of Cu, S and Mo elements for the sample grown at 200 °C. The white lines on the figure depict the actual MoS_2_/sapphire and Cu/MoS_2_ interfaces.

To further investigate the influence of growth temperatures to the morphologies and crystallinity of the thin Cu films, two more samples with 15 nm Cu deposited on MoS_2_ surfaces at 100 and 200 °C are prepared. The 1 × 1 $\upmu$m^2^ AFM images of the two samples are shown in Fig. 2a. The 1 × 1 $\upmu$m^2^ AFM image of the sample prepared at RT is also shown in the figure. With increasing growth temperatures, significant Cu grain coalescence is observed. Flattened plateau with deeper trenches in between is also observed at higher growth temperatures. The phenomenon of Cu grain coalescence will also result in an increasing RMS roughness values from 1.04, 1.44 to 2.23 nm for the Cu films grown at RT, 100 and 200 °C, respectively. The results may indicate improved crystallinity of the thin Cu films with increasing growth temperatures. The normalized Cu (111) XRD peaks of the three samples are shown in Fig. 2b. As shown in the figure, the full widths at the half maximum (FWHM) of the Cu (111) XRD peak decreases from 0.69, 0.64 to 0.61 degree for the three samples prepared at RT, 100 °C and 200 °C, respectively, which suggests that improved crystallinity/larger Cu grains are obtained with increasing growth temperatures. The results are consistent with the observation of enlarged Cu grains with increasing growth temperatures from the AFM images. However, although improved crystallinity is obtained at higher growth temperatures, the resistivity values of the thin Cu films will increase from 6.07 (RT), 8.16 (100 °C) to 40.2 (200 °C) μΩ-cm with increasing growth temperatures. The less continuous Cu films resulted from the Cu grain coalescence with increasing growth temperatures may be the main mechanism responsible for this phenomenon. Since the role of MoS_2_ is to replace the Ta/TaN stack in interconnects, its effect to block the Cu diffusion is a critical issue for this purpose. The cross-sectional high-resolution transmission electron microscope (HRTEM) images of the three samples are shown in Fig. 2c. As shown in the figure, clear tri-layer MoS_2_ with polycrystalline Cu films are observed for the two samples prepared at RT and 100 °C, respectively. However, for the sample prepared at 200 °C, the Cu/MoS_2_ interfaces are less clear. Some MoS_2_ layers seems to be lifted away from the sapphire substrates, which may indicate Cu diffusion into the MoS_2_ films at 200 °C. To investigate this phenomenon, the high-angle annular dark field (HAADF) mappings of Cu, S and Mo elements for the sample grown at 200 °C are shown in Fig. 2d. The observation of Cu signals in the MoS_2_ layer suggests that Cu atoms will diffuse into MoS_2_ at 200 °C, which is also observed for the sample grown at 100 °C. The HAADF mappings of Cu, S and Mo elements for the sample grown at 100 °C are shown in the supplementary information Fig. S1. As shown in Fig. 2c, the diffused Cu atoms may lift some MoS_2_ layers away from the sapphire substrates. In this case, both Mo and S signals will also be observed in the Cu layer near the Cu/MoS_2_ interface.

### Copper films grown on WSe_2_ surfaces

**Figure 3:**
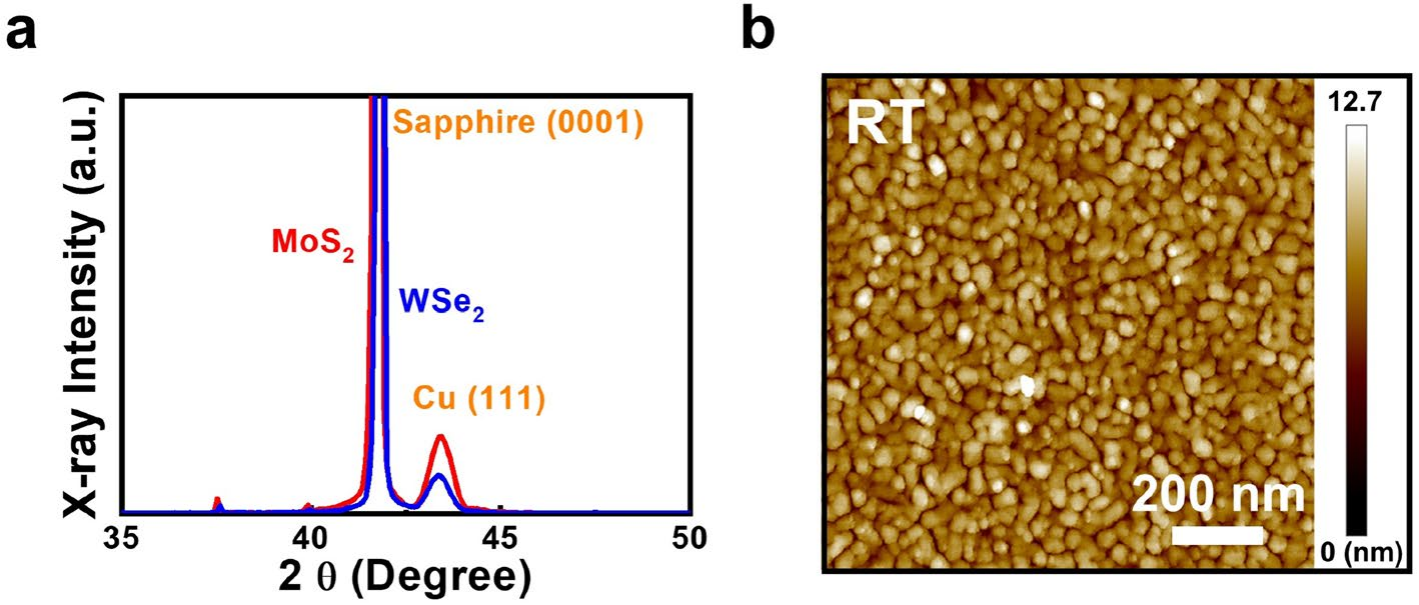
(**a**) The 2θ–θ curve measured by XRD and (**b**) the 1 × 1 $\upmu$m^2^ AFM image of the 15 nm Cu grown on the WSe_2_ surface. The X-ray curve of the Cu film grown on the MoS_2_ surface at RT is also shown in (**a**) for comparison.

Since Cu atoms will easily react with sulfur, it is possible that the defects with Mo vacancies will induce Cu diffusion into the MoS_2_ layers. The element sulfur is also a potential contaminant for semiconductor fabrication lines. In this case, a non-sulfur 2D material should be adopted for the application of liner/barrier layer in interconnects. To demonstrate this point, wafer-scale tri-layer tungsten diselenide (WSe_2_) films are grown on sapphire substrates by selenizing pre-deposited tungsten films at 950 °C. The picture and the cross-sectional HRTEM image of the standalone WSe_2_ sample showing its uniformity are shown in the supporting information (Fig. S2). By using the WSe_2_/sapphire samples as the new substrates, a 15 nm Cu film is deposited on the WSe_2_ surface at RT. The 2θ–θ curve of the sample measured by XRD is shown in Fig. 3a. The X-ray curve of the Cu film grown on the MoS_2_ surface at RT is also shown in the figure for comparison. Besides the substrate peak, the Cu (111) peak is observed on both MoS_2_ and WSe_2_ samples. The results indicate that compared with the Cu film grown on the SiO_2_ surface (Fig. 1), better crystallinity is obtained for the thin Cu films grown on both MoS_2_ and WSe_2_ surfaces. The same van der Waals epitaxy of the thin Cu film grown on the MoS_2_ surface may also take place on the WSe_2_ surface. The 1 × 1 $\upmu$m^2^ AFM image of the sample grown on the WSe_2_ surface is shown in Fig. 3b. Compared with the sample grown on the MoS_2_ surface (Fig. 2a), a similar morphology is observed for the sample grown on the WSe_2_ surface, which indicates that the unique van der Waals epitaxy growth mode will take place on different 2D material surfaces. The preferential planar film growth instead of island formation will improve the continuity of the thin Cu films deposited on both MoS_2_ and WSe_2_ surfaces. In this case, a similar resistivity value 6.66 μΩ-cm with the sample grown the MoS_2_ surface (6.07 μΩ-cm) will be observed for the 15 nm Cu film grown on the WSe_2_ surface.

**Figure 4:**
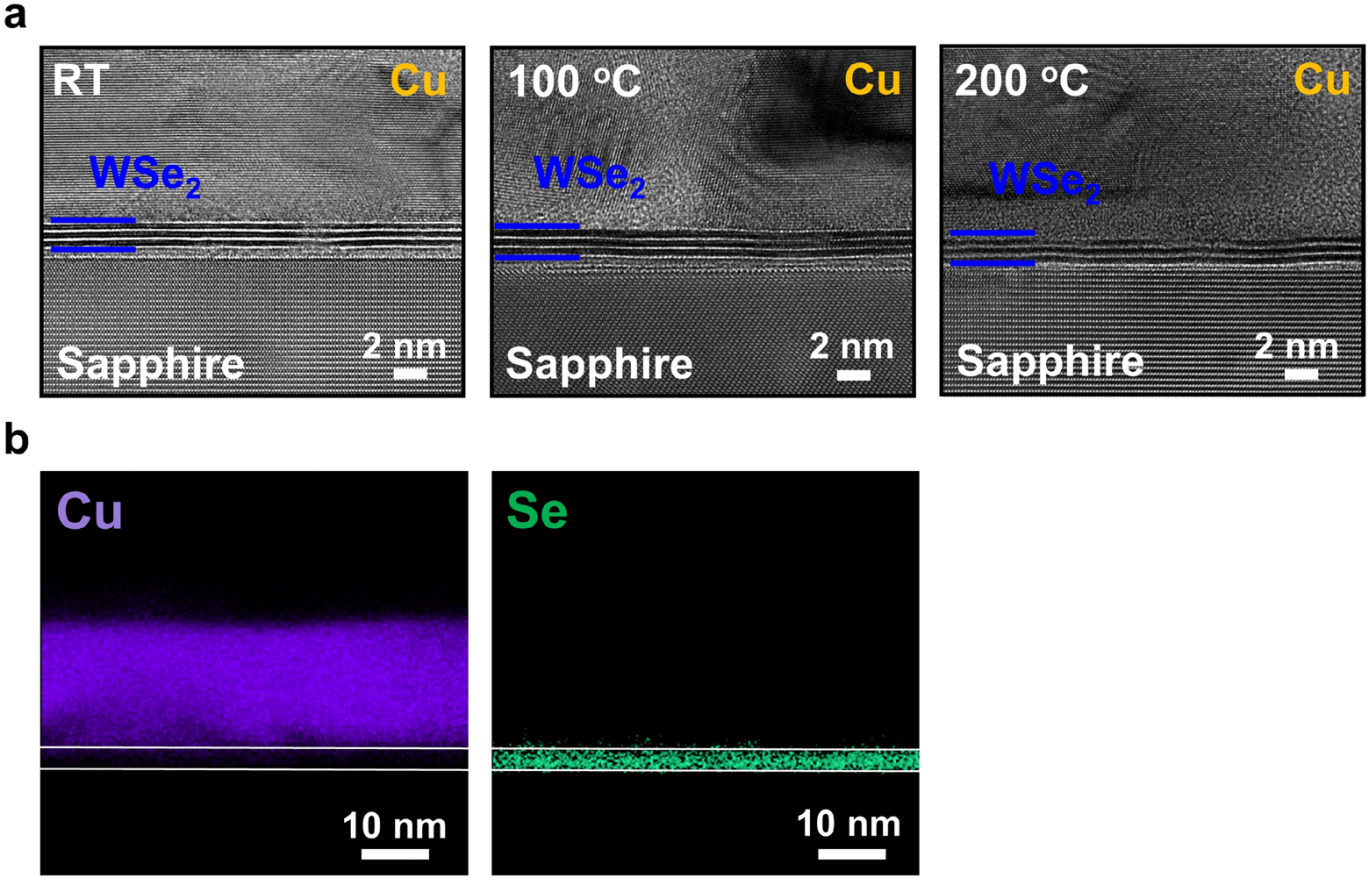
(**a**) The cross-sectional HRTEM images of 15 nm Cu deposited on tri-layer WSe_2_/sapphire substrates at RT, 100 and 200 °C. (**b**) The HAADF mappings of Cu and Se elements for the sample grown at 200 °C. The white lines on the figure depict the actual WSe_2_/sapphire and Cu/WSe_2_ interfaces.

To investigate the influence of growth temperatures to the Cu diffusion in the WSe_2_ samples, two more samples with 15 nm Cu deposited on WSe_2_ surfaces at 100 and 200 °C are prepared by using the e-beam deposition system with 0.1 Å/s deposition rate. Similar with the samples grown on MoS_2_ surfaces, Cu grain coalescence and decreasing FWHMs of the Cu (111) XRD peak are observed for the WSe_2_ samples with increasing growth temperatures (Fig. S3 in the supporting information). The results confirm again that the same van der Waals epitaxy occurs on both MoS_2_ and WSe_2_ surfaces. The cross-sectional HRTEM images of 15 nm Cu films grown on WSe_2_ surfaces at RT, 100 and 200 °C are shown in Fig. 4a. Unlike partial MoS_2_ liftoff from the sapphire substrates for the sample grown on the MoS_2_ surface at 200 °C, polycrystalline Cu films with clear tri-layer WSe_2_ are observed for all the three samples. The results suggest that WSe_2_ layers may effectively prevent Cu diffusion. The HAADF mappings of Cu and W elements for the sample grown at 200 °C are shown in Fig. 4b. A clear separation of Cu and Se signals are observed in the figure. The results indicate that by using non-sulfur 2D materials as the barrier layer, Cu diffusion can be effectively avoided. Compared with the ~ 4 nm thick Ta/TaN stacks, the thickness of tri-layer WSe_2_ is around 2.0 nm. Further thickness reduction is possible by reducing the layer numbers of the WSe_2_ films. The thinner line/barrier stacks of interconnects will leave a large volume for Cu films such that further size reduction is possible for the interconnects. The relatively low resistivity values 6.07 and 6.66 μΩ-cm and the continuous Cu films observed on MoS_2_ and WSe_2_ surfaces at RT suggest a good wettability of Cu films on 2D material surfaces. The thin body nature, the capability to prevent Cu diffusion and the unique van der Waals epitaxy growth mode of 2D materials will make WSe_2_ a promising candidate to replace the Ta/TaN stack in interconnects with reducing linewidths.

**Figure 5:**
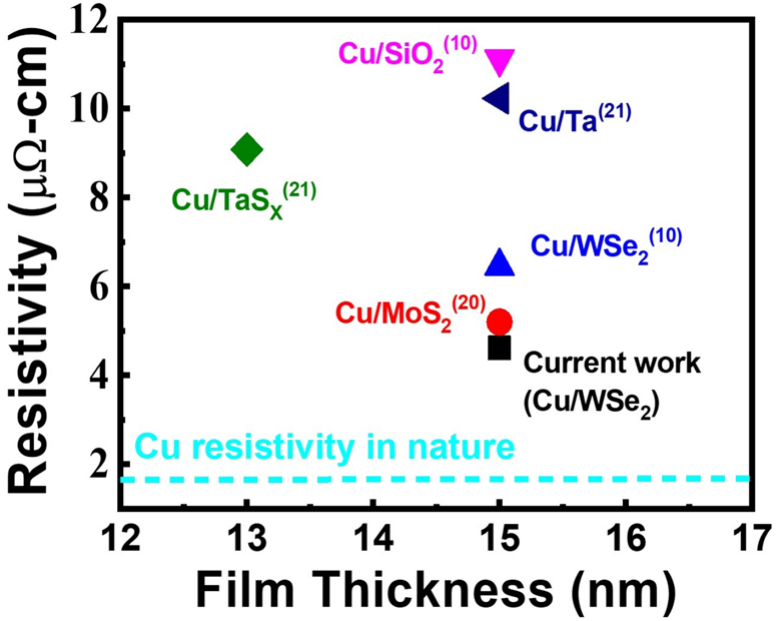
The resistivity values of thin Cu films prepared by using different approaches and subtracts in literature. The resistivity value of the 15 nm Cu film deposited on the WSe_2_ surface at RT in this work is also shown in the figure. The dashed curve shows the resistivity value of Cu in nature (1.68 μΩ-cm).

Although the van der Waals epitaxy growth mode on 2D material surfaces is advantageous for planar growth of metal films. The polycrystalline nature of grown 2D materials with wafer scale may hinder the adatom migration and induce local island formation on the 2D material surfaces. In this case, compared with exfoliated 2D material flakes, higher resistivity values will be observed on grown 2D material surfaces with the same metal film thicknesses. As discussed in the previous section, although a resistivity value 6.07 μΩ-cm can be achieved for the 15 nm Cu film grown on the MoS_2_ surface at RT, a lower resistivity value ~ 5 μΩ-cm is still observed on exfoliated MoS_2_ flakes with the same Cu thickness^20^. As we have observed for the samples with different growth temperatures, the Cu grain coalescence will improve the crystallinity of the film. The same mechanism will also induce a less continuous film such that higher resistivity values will be observed. Therefore, one possible approach to solve this problem is to increase the adatom density on the substrate surfaces such that a more continuous film can be obtained. 15 nm Cu films with deposition rates 0.5 and 1.0 Å/s are deposited on WSe_2_ surfaces by using the e-beam deposition system. The resistivity values obtained for the two samples are 5.22 and 4.62 μΩ-cm, respectively. The resistivity values of Cu films reported in previous publications are shown in Fig. 5. The resistivity value 1.68 μΩ-cm of Cu in nature is also shown in the figure. As shown in the figure, the resistivity value 4.62 μΩ-cm is the lowest value reported in literature. Given the fact that polycrystalline instead of single-crystal films are obtained for wafer-scale 2D materials at this stage, it is possible to achieve further reduction in the resistivity value for the thin Cu films if improved crystallinity can be achieved for grown 2D materials in the future. Nevertheless, the recorded low resistivity value of the thin Cu film grown on the WSe_2_ surface has already demonstrated its potential to replace the Ta/TaN stack in interconnects. Besides the direct growth of 2D materials on different substrates, the film transferring can be an easy approach to facilitate the integration of 2D materials with other substrates. After transferring the tri-layer WSe_2_ to a 300 nm SiO_2_/Si substrate, 15 nm Cu is deposited on the WSe_2_ surface by using the e-beam deposition system with the deposition rate 1.0 Å/s at RT. Compared with the value 4.62 μΩ-cm obtained for the sample grown on the un-transferred WSe_2_ surface, a similar resistivity value 5.43 μΩ-cm is observed for this sample. Despite the contamination and damages introduced during the transferring procedure, the results have demonstrated that van der Waals epitaxy is still the main mechanism of epi-layer growth on 2D material surfaces.

## Conclusion

We have demonstrated 15 nm Cu film deposited on different 2D material surfaces. With the assist of van der Waals epitaxy growth mode on 2D material surfaces, preferential planar growth is observed for Cu films on both MoS_2_ and WSe_2_ surfaces at room temperature, which will induce a polycrystalline and continuous Cu film formation. At higher growth temperature 200 °C, Cu diffusion into the MoS_2_ layers is observed while the non-sulfur 2D material WSe_2_ can prevent Cu diffusion at the same growth temperature. By further increasing the deposition rates, a record-low resistivity value 4.62 μΩ-cm for thin Cu films is observed for the sample grown on the WSe_2_ surface. After transferring the tri-layer WSe_2_ to a 300 nm SiO_2_/Si substrate, a similar resistivity value 5.43 μΩ-cm is obtained with 15 nm Cu deposited on the WSe_2_ surface. The low resistivity values of thin Cu films grown on transferred or un-transferred WSe_2_ surfaces have provided varieties for the 2D material integration with other materials in practical applications. These results have demonstrated that non-sulfur 2D materials such as WSe_2_ can be a promising candidate to replace the liner/barrier stack in interconnects with reducing linewidths.

## Methods

For the growth of tri-layer MoS_2_, the Mo metal was deposited on the sapphire substrates by using a radio-frequency (RF) sputtering system. We used 30 W for constant sputtering power and 5 × 10^−3^ Torr with a 30 sccm gas flow of argon (Ar) for background pressure. The deposition time is 37 s. for the preparations of MoS_2_ layer. After the metal deposition, the sample was placed at the center of the hot furnace for sulfurization. 200 sccm Ar gas was used as carrier gas while the pressure was kept at 50 Torr. The sulfurization temperature was kept at 850 °C with 0.25 g of sulfur (S) powder for 20 min. For the growth of tri-layer WSe_2_, a similar transition metal deposition and selenization procedure is adopted. The W metal film was deposited on the sapphire substrate by using the same RF sputtering system. Similar deposition conditions as the Mo deposition are adopted for the W deposition except for the higher sputtering power 40 W. The deposition time is 25 s. for the W deposition. After the metal deposition, the sample was placed at the center of the hot furnace for selenization. 85 sccm Ar gas was used as carrier gas while the pressure was kept at 100 Torr. The selenization temperature was kept at 950 °C with 0.2 g of Se powder for 30 min. The deposition rate and the thickness of the Cu films are measured by the quartz crystal microbalance (QCM) which is equipped with the e-beam system. The resistivity values were obtained through the equation $\rho$ = R_S_ × (film thickness), where R_S_ is the sheet resistance obtained through four-point measurements. For the four-point measurements, a four-pin probe station KSR-4 with BeCu probe tip and tip pitch 1 mm is adopted. Keithley 2400 SourceMeter is used to measure the sheet resistance of the samples. The cross-sectional HRTEM images measurement are obtained by using a JEOL JEM-2800F transmission electron microscopy system operated at 200 kV. The XRD measurement are done by using a Bruker New D8 Discover XRD system with Cu Kα radiation (λ = 1.5406 Å). The AFM measurement are done by using a BRUKER Dimension ICON AFM system.

## Supplementary Information

Supplementary Information.
