# Supplementary material for: Nanometer-thick copper films with low resistivity grown on 2D material surfaces
Source: Sci Rep. 2022 Feb 2;12:1823. doi: 10.1038/s41598-022-05874-9 (PMC8810854; doi:10.1038/s41598-022-05874-9)
Supplement: Supplementary file 1 — Supplementary Information. [file 41598_2022_5874_MOESM1_ESM.pdf]

Supplementary Information

Nanometer-thick Copper Films with Low Resistivity Grown on 2D Material Surfaces

Yu-Wei Liu[1], Dun-Jie Zhang[1, 2], Po-Cheng Tsai[1, 3], Chen-Tu Chiang[1], Wei-Chen Tu[2], and Shih-Yen Lin[1, 3 *]

[1]Research Center for Applied Sciences, Academia Sinica, No. 128, Sec. 2, Academia Rd., Taipei 11529, Taiwan

[2]Department of Electrical Engineering, National Cheng Kung University, No.1, University Road, Tainan City 701, Taiwan

[3]Graduate Institute of Electronics Engineering, National Taiwan University, No. 1, Sec. 4, Roosevelt Rd., Taipei 10617, Taiwan

*Corresponding author, electronic mail: shihyen@gate.sinica.edu.tw

The HAADF mappings of Cu, Mo and S elements for the 15 nm Cu film deposited at 100 °C on the MoS_2 surface are shown at Fig. S1. As shown in the figure, we have observed minor Cu down diffusion to the MoS_2 layer. However, no significant MoS_2 lift from the sapphire surface as observed for the sample grown at 200 °C is observed for this sample.

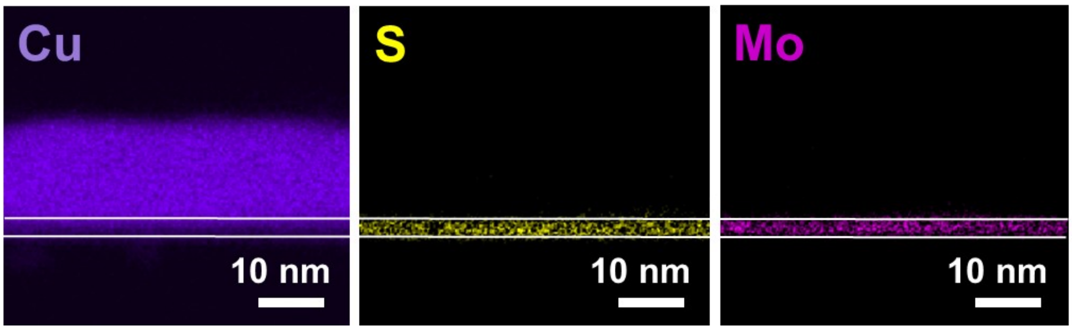

Figure S1 The HAADF mappings of Cu, S and Mo elements for the sample with a 15 nm Cu film deposited on the MoS_2 surface at 100 °C. The white lines on the figure depict the actual MoS_2/sapphire and Cu/MoS_2 interfaces.

The picture and the Raman mappings of the tri-layer WSe_2 grown by selenizing pre-deposited W films are shown in Figure S1a and b, respectively. As shown in Fig. S1 (a), wafer-scale WSe_2 is obtained after the selenization of pre-deposited W films. However, unlike the increasing energy difference of MoS_2 Raman peaks with increasing layer numbers, the energy difference between E^1_{2g} and A_{1g} Raman peaks of WSe_2 will decrease with increasing layer numbers. The energy difference is also small for WSe_2. Therefore, it is difficult to tell the approximate layer numbers directly from

the Raman spectrum. Nevertheless, through the observation of cross-sectional HRTEM image of the standalone WSe_2/sapphire sample with smaller magnification shown in Figure S1c, a wafer-scale WSe_2 film with good layer number uniformity can be observed by using this growth approach.

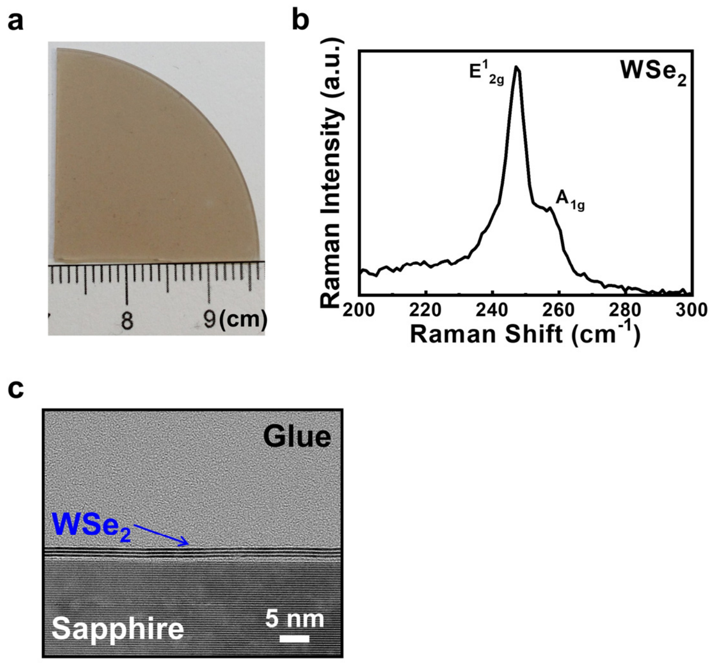

Figure S2. (a) The picture, (b) the Raman mappings and (c) the cross-sectional HRTEM image with smaller magnification of the tri-layer WSe_2 grown by selenizing pre-deposited W films.

Similar with the samples grown on MoS_2 surfaces, decreasing FWHMs of the Cu (111) XRD peak are observed for the WSe_2 samples with increasing growth temperatures. The analysis to the FWHMs of Cu (111) XRD peak for the samples grown on WSe_2 surfaces at different temperatures are shown in Fig. S2.

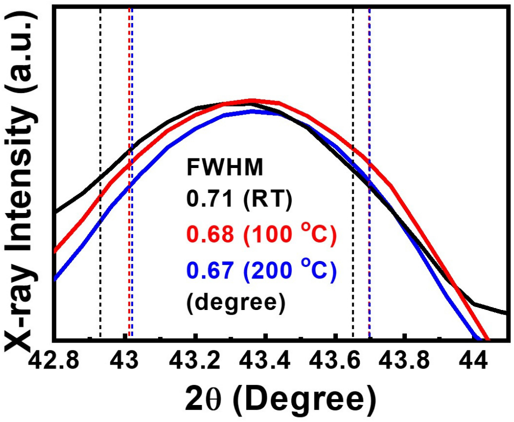

Figure S3. The FWHMs of Cu (111) XRD peak for the samples grown on WSe_2 surfaces at RT, 100 and 200 ^{o}C.
